# Targeting SUMOylation triggers interferon-β-dependent activation of patient and allogenic Natural Killer cells in preclinical models of Acute Myeloid Leukemia

**DOI:** 10.1158/1535-7163.MCT-25-0504

**Published:** 2026-01-02

**Authors:** Rawan Hallal, Marion de Toledo, Denis Tempé, Rayane Berrahouane, Sara Zemiti, Loïs Coënon, Delphine Gitenay, Simon George, Moritz Schüssler, Nadine Laguette, Sarah Bonnet, Ludovic Gabellier, Guillaume Cartron, Mireia Pelegrin, Martin Villalba, Guillaume Bossis

**Affiliations:** 1 Equipe Labellisée Ligue contre le Cancer, IGMM, Univ Montpellier, CNRS, Montpellier, France; 2 Service d’Hématologie Clinique, CHU de Montpellier, 80 avenue Augustin Fliche, 34091 Montpellier, France; 3 IRMB, Univ Montpellier, INSERM, CHU Montpellier, Montpellier, France; 4 MGX, Univ Montpellier, CNRS, INSERM, Montpellier, France; 5 IGMM, Univ Montpellier, CNRS, Montpellier, France

## Abstract

Natural Killer (NK) cells can play a significant role in the anti-tumoral immune response. In patients with Acute Myeloid Leukemia (AML), NK cells are however often found in low numbers and exhibit poor activity, contributing to leukemic progression. Allogenic NK cells are emerging as promising cellular therapies for hematological cancer treatment. New strategies are however required to both reactivate NK cells in AML patients and enhance the anti-tumor activity of transplanted NK cells. Here, we demonstrate that targeting SUMOylation, a protein post-translational modification, activates NK cells from both healthy donors and AML patients. Subasumstat (TAK-981), a first-in-class inhibitor of SUMOylation used in phase I/II clinical trials, enhances NK cells degranulation, secretion of inflammatory cytokines (IFN-γ, TNF-α, FasL) and cytotoxicity against AML cells. *In vivo*, TAK-981 improves the anti-leukemic efficacy of *ex-vivo* expanded cord-blood NK cells in leukemia-bearing mice. One early effect of TAK-981 is to specifically increase the accessibility and activation of cis-regulatory regions of type I interferon (IFN-I) pathway genes and induce their transcription. TAK-981-induced secretion of interferon-β, mostly by NK cells and myeloid cells, is required for NK cells activation. Surprisingly, *IFNB1* induction does not require its best-characterized activators MDA5, cGas, IRF-1, -3 and -7. Altogether, this suggests that targeting SUMOylation activates a non-canonical IFN-I pathway, which enhances the anti-leukemic potential of NK cells.

## Introduction

Acute Myeloid Leukemias (AML) are a group of aggressive myeloproliferative malignancies with poor prognosis. High numbers of circulating NK cells and higher NK cell cytotoxicity correlate with better prognosis in AML patients, lending support to the hypothesis that NK cells themselves could potentially significantly participate in controlling AML progression (1). However, frequent deficiencies in NK cells number and activity facilitate AML cells escape from immune surveillance (2). NK cells are also increasingly considered as a promising cellular therapy for cancer, including for AML. This includes adoptive transfer of *ex vivo* expanded NK cells and chimeric antigen receptor-modified NK cells (CAR-NK)(3–5). NK cells, whether endogenous or allografted, recognize cancer cells (i) with downregulated MHC class I via inhibitory receptor disengagement and (ii) expressing stress-induced ligands, which bind NK activating receptors (e.g. NKG2D, DNAM-1)(6). One of the main interests of allogenic NK cells transplantation lies in their potential to eliminate tumor cells without causing graft-versus-host disease (GVHD), as they do not express antigen-specific receptors like T-Cell (7). However, new strategies are required to enhance their activation and thus strengthen their anti-tumoral activity (8–10).

SUMOylation is a post-translational modification, which consists in the covalent conjugation of SUMO-1, -2 or -3 on lysines of thousands of proteins to control their function and fate (11). One major role of SUMOylation is the control of gene expression through the modification of various transcription factors and co-regulators (12,13). We and others have recently shown that Subasumstat (TAK-981)(14), a first-in-class inhibitor of the SUMO E1 activating enzyme, has potent anti-leukemic activity in AML preclinical models (15,16).

Recent studies have reported that targeting SUMOylation activates an anti-tumor immune response and improves the efficiency of immune therapies by directly affecting immune cells (17–22). In particular, TAK-981 was found to enhance the antitumoral activity of monoclonal antibodies such as rituximab (anti-CD20) or daratumumab (anti-CD38) in preclinical mouse models of lymphoma and myeloma respectively (18). These combination therapies were evaluated in two clinical trials (clinicaltrials.gov, accession numbers NCT04776018 and NCT04074330). TAK-981 also increases the anti-leukemic efficacy of TCR-engineered CD8 T-cells used for adoptive transfer in preclinical models of leukemias (23). The immuno-modulatory function of TAK-981 was suggested to rely on its ability to induce type-I Interferon (IFN-I) secretion, which activates both innate and adaptative anti-tumoral immune response (17–22). Contrasting with these results, TAK-981-dependent activation of T-cells from Chronic Lymphocytic Leukemia patients was found independent of IFN-I signaling (24).

Here, we demonstrate the potential of TAK-981 to both restore the anti-leukemic activity of AML patients’ NK cells and increase, *in vivo*, the cytotoxicity of *ex vivo* expanded cord-blood NK cells. TAK-981 induces a strong increase in NK cells activation, degranulation capacity and inflammatory cytokines secretion. This enhances their cytotoxicity towards AML cell lines and patient blasts. At the molecular level, we demonstrate that the main effect of TAK-981 is a rapid activation of enhancers of IFN-I pathway genes. TAK-981 induces IFN-I secretion, leading to an autocrine activation of various ISGs and subsequent activation of NK cells. In addition, NK cells are activated *in trans* by IFN-I secreted mostly by myeloid cells upon TAK-981 treatment.

## Methods

### Ethics approval

Buffy coats were obtained from healthy blood donors from the Etablissement Français du Sang (EFS, agreement n°21PLER2019-0002). Bone marrow aspirates and blood samples were collected from AML patients, after obtaining written informed consent under the frame of the Declaration of Helsinki and after approval by the Institutional Review Board (Ethical Committee "Sud Méditerranée 1," ref 2013-A00260-45, HEMODIAG_2020 cohort). All experiments on animals were approved by the Ethics Committee of the Languedoc-Roussillon (2018043021198029 #14905 v3).

### Myeloid and NK cells purification

PBMC were purified using density-based centrifugation (Histopaque 1077- H8889 Sigma-Aldrich). Myeloid cells were purified from PMBC using CD33+ microbeads (Miltenyi 130-045-501). For NK cells, PBMCs depleted from CD33+ cells, were purified using negative selection NK cells isolation Kit (Miltenyi 130-092-657). The purity of NK cells was assessed by flow cytometry with CD56 and CD3 marker (CD56^+^/CD3^-^). Only samples with a purity above 95% were used.

### FACS sorting

PBMC, labelled with conjugated antibodies (Supplementary Table S1) were sorted using a BD FACS Aria IIu Cell sorter. The following markers were used: NK cells: CD56+/CD3-; NKT cells: CD56+/CD3+; CD8 T lymphocytes: CD3+/CD8+; CD4 T lymphocytes: CD3+/CD4+; B lymphocytes: CD3-/CD19+; Monocytes HLA-DR+/CD14+; Conventional dendritic cells (cDC): HLA-DR+/CD14-/CD11c+; Plasmacytoid dendritic cells (pDC): HLA-DR+/CD14-/CD11C-/CD123+.

### Cell culture

Human AML cell lines U937-LucZsGreen and THP-1-LucZsGreen expressing both luciferase and ZsGreen protein were cultured as described (15). U937 (male patient) were a gift from Dr JE.Sarry in 2011 and THP-1 (male patient) from Dr D.Mathieu in 2010. Cell line authentication was performed on 29/12/2022 by SPR-profiling (Eurofins) for parental U937 and THP-1. Cells were used for a maximum of 10 passages after thawing and regularly tested (last testing 2025-03-06) for Mycoplasma contamination (Lonza, #LT07-318). IFN-α/β Reporter HEK 293 cells were obtained from InvivoGen and cultured according to manufacturer protocol.

### eNK amplification from cord blood

eNK expansion protocol was adapted from a previously described protocol (25) using umbilical cord blood (UCB) (biobank BB-0033-00031 from CHU Montpellier). CD3 positive cells were removed from UCB mononucleated cells using EasySep™ Human CD3 Positive Selection Kit II (Stemcell). CD3 negative cells were cultured at 1×10^6^ cells/mL in eNK medium (RPMI1640 Glutamax™ or NK MACS, Miltenyi) supplemented with 10% FBS or 5% human serum, 100 IU/mL rhIL-2 (Peprotech) and 5 ng/mL hrIL-15 (Miltenyi). From day 5-7 and every 2-3 days until day 14-21, medium was removed and freshly prepared eNK medium added to reach 0.6×10^6^ cells/mL. 70 Gy-irradiated lymphoblastoid B-EBV feeder cells were added at appropriate ratios. eNK cells phenotype was analyzed by flow cytometry at day 14 on a Gallios flow cytometer (Beckman Coulter) after labelling with 7AAD (Viability), anti-CD45-VioGreen, anti-CD19-APC-Vio770, anti-CD56-PE-Vio770, anti-CD69-PE, anti-CD16-VioBlue, anti-CD3-APC (Supplementary Table S1).

### Pharmacological inhibitors and survival analysis

TAK-981 was obtained from Takeda Development Center Americas, Inc. TAK-981 was re-suspended in DMSO at 10 mM and used at 100 nM. IFNAR2 neutralizing monoclonal antibody (MMHAR2 Sigma-Aldrich 407295) was used at 1 µg/mL. Cell survival was measured using flow cytometry based on FSC and SSC parameters for total PBMCs, and CD56 and CD3 markers to identify NK cells.

### NK cells cytotoxicity assay

Purified NK cells or total PBMC were co-cultured with AML target cells (AML patient’s blasts, U937-LucZsGreen or THP-1-LucZsGreen) at a ratio effector:target cells of 1:1 for NK cells or 10:1 for PBMC. AML patient’s blasts were stained with 1 µM CFSE (eBioscience-65-0850-85) prior to co-culture. Four hours after co-culture, NK cells activity was measured using CD69 and CD107a markers and the total number of green fluorescent AML cells was counted.

For real time killing assays, purified NK cells or AML patients PBMCs were co-cultured with U937-LucZsGreen or THP-1-LucZsGreen. The number of fluorescent AML cells was assessed using the IncuCyte S3 Live Cell imaging system (Sartorius) in the non-adherent cell-by-cell mode 20X objective, 9 images/well and 1 image/hour. Analyses were performed by measuring the relative integrated green fluorescence intensity as a proxy for the number of AML cells using IncuCyte 2022A software.

### Flow cytometry

For NK cells degranulation assay, CD107a antibody was added to the cells at the beginning of the co-culture. One hour later, Monensin sodium salt (Sigma-Aldrich M5273) was added at 6 µg/ml to block exocytosis. Cells were then incubated for 3 h and analyzed with NovoCyte Flow Cytometer (Agilent). For the other markers, cells were washed once with PBS, incubated 15 min at room temperature in PBS 5% FBS with conjugated antibodies (Supplementary Table S1), washed with PBS and analyzed with NovoCyte Flow Cytometer (Agilent) and NovoExpress software (v.1.5.6).

### Cytokines quantification

Supernatants were collected and cytokines were quantified using Human CD8/NK panel (LEGENDplex 740267) according to manufacturer guidelines. IFN-I was quantified using HEK-Blue™ IFN-α/β reporter cells (InvivoGen - hkb-ifnabv2). Cells were seeded at 30.000 cells/well in 96-well plate. After 24 h, cell culture supernatants were added to the reporter cells and incubated overnight. SEAP activity was then quantified using Quanti-Blue (InvivoGen, rep-qbs).

### Transwell assay

PBMC were placed in the upper chamber of transwell inserts with 0.4 µm pore size (Falcon - 353095) and NK cells were placed in the lower chamber. NK cells were collected 24 h later and co-cultured with U937-LucZsGreen cells for 4 h. CD69 expression on NK cells and AML cells survival were analyzed by flow cytometry.

### *In-vivo* mice models

6-10 weeks old Fox chase mice (CB17/Icr-Prkdc^scid^/IcrIcoCrl) were treated IV with TAK-981 (15 mg/kg) resuspended in 20% HPBCD (hydroxypropyl beta-cyclodextrin) or vehicle (20% HPBCD). Intraperitoneal wash was performed by injecting 5 mL of PBS + 3% FBS into the peritoneal cavity of the mice followed by recollecting the PBS. Total cells collected were labeled with anti-CD3, anti-CD69 and anti-NKp46 antibodies (Supplementary Table S1) and analyzed by flow cytometry. For experiments comparing WT and IFNARKO C57BL/6 mice, blood, spleen and bone marrow (from femurs) were collected and cells were dissociated and washed with PBS. Red blood cells were lysed using the ACK lysis buffer (A1049201, Gibco) and mononuclear cells were labeled with anti-CD11b, anti-CD19, anti-CD69, anti-CD3, anti-CD45, anti-CD122 antibodies (Supplementary Table S1) and analyzed by flow cytometry. Mice NK cells were gated as CD45+/CD11b- SSC low/CD3-/CD19-/CD122+ (26), and NK cells activation level was measured using Median fluorescent Intensity (MFI) of CD69.

For Cell Line Derived Xenograft (CLDX), 6-10 weeks old female NOD/LtSz-SCID/IL-2Rγchain null (NSG) mice (Charles River) were treated IV with 30 mg/kg busulfan (SIGMA B2635). Two days after, mice were injected IV with 10^6^ THP-1-LucZsGreen cells resuspended in PBS. Mice were then randomized according to luminescence intensity. No blinding was performed. Mice were then injected with 10^7^ expanded NK cells (eNK) at day 7 and 10 post THP-1 injection. Mice were then treated with TAK-981 (IV, 15 mg/kg) and IL-15 (IP, 0.25 µg/mice, Miltenyi Biotech 130-095-760) twice a week.

### RNA-seq libraries preparation and sequencing

Total RNA was purified using GeneElute Single Cell RNA Purification Kit (Sigma-Aldrich RNB300), treated with DNase-I on column (Sigma- DNASE70-1SET). RNA quality was checked using a BioAnalyser Nano 6000 Chip (Agilent). Libraries were prepared using SMART-Seq Stranded Kit (Takara Bio) and sequenced using an Illumina Novaseq 6000 sequencer as single-end 100 base reads. RNA-seq analysis was performed using FastQC and Cutadapt for adapter trimming, then aligned to the reference genome using HISAT2. Differential gene expression analysis was conducted using edgeR (27) with trimmed mean of M-values (TMM) paired normalization. Genes with a fold change >2 or < 0.5 and an adjusted p-value < 0.05 were considered differentially expressed. Gene Set Enrichment Analyses were performed using https://www.gsea-msigdb.org/gsea/index.jsp (version 4.0.3)(28).

### CUT&RUN

500,000 cells per condition were harvested by centrifugation, washed once with PBS and resuspended in 100 µL PBS. BioMag Plus Concanavalin A beads (Polysciences #86057) were equilibrated in activation buffer (20 mM HEPES pH7.5, 150 mM NaCl, 0.5 mM Spermidine, 0.1% Digitonin, 1X Protease Inhibitors (EDTA-free)) and incubated with the harvested cells for 15 min at room temperature. Cell-bead complexes were collected with a magnet and resuspended in 100 µL of antibody solution containing anti-H3K27Ac (ABclonal A7253) or Rabbit IgG (Millipore 12-370) diluted at 1:100 in wash-digest buffer (20 mM HEPES pH7.5, 150 mM NaCl, 0.5 mM Spermidine, 0.1% Digitonin, 1X Protease Inhibitors (EDTA-free)). After overnight incubation with the antibodies at 4°c, the cell-bead complexes were collected and wash-digest buffer supplemented with pA/G-MNase was added. Cell-bead mixture was incubated for 2 h at room temperature on a rotator. Cells-bead complexes were then washed once with low salt buffer (20 mM HEPES pH 7.5, 0.5 mM spermidine, 0.1% Digitonin). Chromatin digestion was initiated by resuspending the cell-bead complexes in 100 µL incubation buffer (3.5 mM HEPES pH 7.5, 10 mM CaCl_2_, 0.1% Digitonin) on ice for 30min. The reaction was stopped by adding 100 µL of 2X-stop buffer (20 mM HEPES pH7.5, 340 mM NaCl, 20 mM EDTA, 6 mM EGTA, 50 μg/ml RNase A, 0.1% Digitonin) and incubating at 37°C for 20 min. Digested DNA fragment were collected and purified using Monarch^®^ PCR & DNA Cleanup Kit (#T1030). Sequencing libraries were generated with the NEBNext® Ultra™ II DNA Library Prep Kit (Illumina #E7645), and purified using cleanNGS beads (CleanNA, #CNGS-0001). Libraries were analyzed by capillary electrophoresis (Fragment Analyzer, NGS HS kit) and sequenced by Illumina Novaseq 6000 sequencer as paired-end 50 base reads. Raw sequencing data were processed using nf-core/cutandrun pipeline (29) with default parameters. Sequencing data were preprocessed using FastQC and TrimGalore, then aligned to the reference genome using Bowtie2. Peak calling was performed using MACS2 in narrow mode with default settings. Differential accessibility and enrichment analyses were conducted using DiffBind with trimmed mean of M-values (TMM) paired normalization in edgeR. Peak annotations identifying the closest genes were generated using the peakAnno R package, and heatmap visualizations were created using deepTools.

### ATAC-seq

ATAC-seq was performed on 100,000 purified NK cells. Cells were washed once with ice cold PBS and lysed with cold lysis buffer (10 mM Tris-HCl, 10 mM NaCl, 3 mM MgCl2 and 0.1% IGEPAL CA-630). Nuclei were harvested by centrifugation and DNA was digested with Tagment DNA Enzyme 1 (Illumina, 15027865 or Diagenode C01070012) for 30 min at 37°C. Tagmented DNA fragments were purified using Monarch PCR and DNA Cleanup Kit (T1030L), and amplified for 11 cycles by PCR using Q5 High Fidelity DNA Polymerase (NEB M0544) and Ad1 and Ad2 primers (Supplementary Table S2). ATAC-seq libraries were sequenced in 50 bp paired-end Illumina sequencing for Replicate 1 and 2 or 100 bp paired-end DNB-Seq sequencing (BGI) for replicates 3 and 4. Raw sequencing data were processed using nf-core/atacseq v2.1.2 pipeline (29) with default parameters. Preprocessing included FastQC quality control, TrimGalore for adapter trimming, and BWA for alignment to the reference genome.

### RT-qPCR assays

Total RNA was purified using TRIzol reagent (Invitrogen -15596026). After DNase I treatment with RQ1 RNase-Free DNase (Promega - M6101), reverse transcription was done using OneScript Plus cDNA Synthesis Kit (ABM- abm-G236). qPCR was performed on 0.2 µg of cDNA using Platinum Taq DNA polymerase (Invitrogen-3644073) and specific DNA primers (Supplementary Table S3). qPCR reaction was analyzed on CFX opus Real-Time PCR device (Bio-Rad) and data were normalized to the expression levels of *GAPDH*.

### Western blot

Equal number of cells were lysed with Laemmli buffer and run on SDS-PAGE. Antibodies against SUMO-1 (21C7), and SUMO2/3 (8A2) were obtained from the Developmental Studies Hybridoma Bank. IRF-1 antibody was from Santa-Cruz (sc-74530), IRF-3 (D83B9) and MDA-5 (D74E4) from Cell Signaling, IRF-7 from ProteinTech (#22392).

### Generation of Knockout Cell Lines

Lentiviral particles were produced by co-transfecting HEK293T cells with the psPAX2 (Addgene #12260) and pCMV-VSV-G (Addgene #8454) plasmids and the lentiviral vector using calcium phosphate precipitation (HBS solution, SIGMA, 51558). Viral supernatants were collected 48 hrs post-transfection, filtered (0.45 µm) and concentrated by ultracentrifugation. The concentrated viral particles were then used to infect THP-1 cells. The THP-1-IRF1 knockout cell line was generated through co-infection of THP-1 cells with lentiviral particles carrying pLentiCas9-Blast (Addgene #52962) and IRF-1 targeting gRNA cloned in Sanger Lentiviral CRISPR vector (Supplementary Table S4). Infected cells were cultured in RPMI medium supplemented with 2.5 µg/mL puromycin and 7.5 µg/mL blasticidin for 3 weeks. For the generation of the THP-1-IRF3, THP-1-IRF7, and THP-1-MDA5 knockout cell lines, THP-1 cells were infected with pLentiCRISPRv2 lentivirus (30) expressing gRNAs targeting IRF3, IRF7 or MDA5 (Supplementary Table S4). Following infection, cells were cultured in RPMI medium supplemented with 2.5 µg/mL puromycin for 3 weeks.

### SEAP-ISRE Reporter Assay

THP1-Dual cells IRF-Lucia luciferase reporter parental, c-GAS KO or IFNAR KO (Invivogen thpd-nfis, thpd-kocgas, thpd-koifnar2 respectively) were used to assess ISRE activity. Cells were seeded at a density of 0.3 × 10^6^ cells/mL one day prior to treatment. Lucia activity was measured using the Quanti-Luc reagent (InvivoGen, rep-qlc4lg1), following the manufacturer’s instructions.

### Statistical analysis

Statistical analyses of the differences between data sets were performed using RM-one-way ANOVA test, except for indicated experiment where paired student t-test was used (GraphPad Prism, GraphPad 10.0.2). Overall mice survival was estimated for each treatment group using the Kaplan-Meier method and compared with the log-rank test. P-values of less than 0.05 were considered significant (*, P < 0.05; **, P < 0.01; and ***, P < 0.001, ns = not significant).

## Results

### SUMOylation inhibition activates NK cells

**Figure 1:**
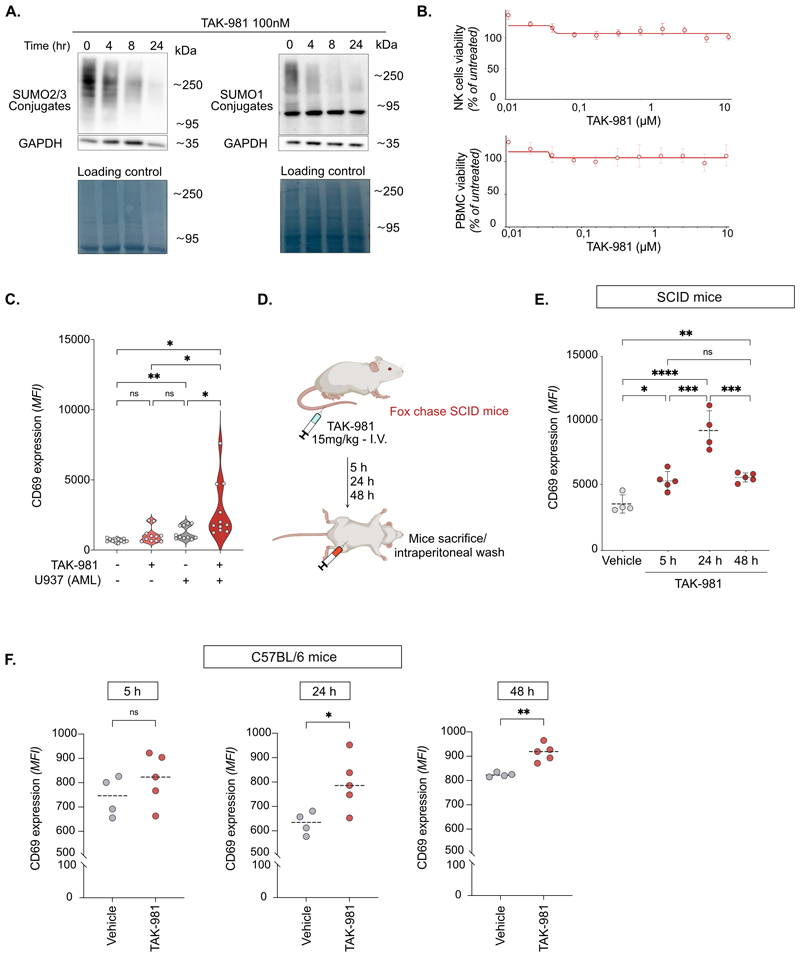
Inhibition of SUMOylation activates NK cells. **A**. Immunoblot with SUMO-2/3 and SUMO-1 antibodies of purified NK cells treated with 100 nM TAK-981 for the indicated times. GAPDH and amidoblack staining were used as loading control. **B**. NK cells (upper panel) or total PBMCs (lower panel) collected from (n=5) healthy donors were treated with increasing concentrations of TAK-981 for 24 h. Cell viability was determined by flow cytometry according to the SSC/FSC profile of the cells, dead cells being SSC^high^/FSC^low^ and living cells being SSC^low^/FSC^high^. A dose-response curve was generated by comparing the viability of TAK-981 treated cells with untreated controls. Data are shown as mean +/-SEM of 5 donors. **C**. Violin plot showing the median fluorescent intensity of CD69 on the surface of purified NK cells. NK cells were treated with 100 nM TAK-981 for 24 h. After removing TAK-981, NK cells were co-cultured with U937 for 4 h (Effector:Target (E:T) ratio 1:1) (n= 10 donors, RM one-way ANOVA test). **D**. Fox chase SCID mice were injected intravenously with 15 mg/kg TAK-981 or vehicle. Mice were euthanized after 5 h, 24 h or 48 h and intraperitoneal cavity wash was performed to collect NK cells. **E**. CD69 expression levels on NK cells from SCID mice (CB17/Icr-Prkdcscid) treated or not with TAK-981 for indicated time (n=4 or 5 mice per group, RM one-way ANOVA). **F**. C57BL/6 mice were injected intravenously with 15 mg/kg TAK-981 or vehicle. Blood was collected after 5 h, 24 h and 48 h and CD69 expression levels on NK cells from blood was analyzed. Data are shown as mean fluorescence intensity (MFI) +/- SD (4 or 5 mice per group, RM one-way ANOVA)

Treatment with the SUMOylation inhibitor TAK-981 of NK cells purified from healthy donors’ blood induced a deconjugation of both SUMO-1 and SUMO-2/3 from their targets, visible after only 4 h of treatment and maximal after 24 h (Figure 1A). TAK-981 had no effect on the viability of purified NK cells (Figure 1B, upper panel) or total PBMC (Figure 1B, lower panel) after 24 h of treatment as measured by flow cytometry (FSC/SSC gating). TAK-981 induced an overexpression of CD69, a marker of lymphocytes activation, on the surface of purified NK cells treated for 24 h and co-cultured for 4 h with U937 AML cell line as target cells (Figure 1C). To confirm the effect of TAK-981 on NK cells activation *in vivo*, we treated SCID mice, which have no T- and B-lymphocytes but have normal NK cells, with TAK-981 (Figure 1D). The activation of NK cells, as measured by the expression of CD69, was already significant after 5 h of treatment, maximal at 24 h and declined after 48 h (Figure 1E). Treatment with TAK-981 of C57BL/6 mice, which are fully immunocompetent, also induced the activation of NK cells, as measured by CD69 expression, in their blood after 24 h and it was still present after 48 h (Figure 1F). Altogether, these data suggested that targeting SUMOylation activates NK cells both *in vitro* and *in vivo*.

### Targeting SUMOylation increases NK cell cytotoxicity against AML cells *in vitro*

**Figure 2:**
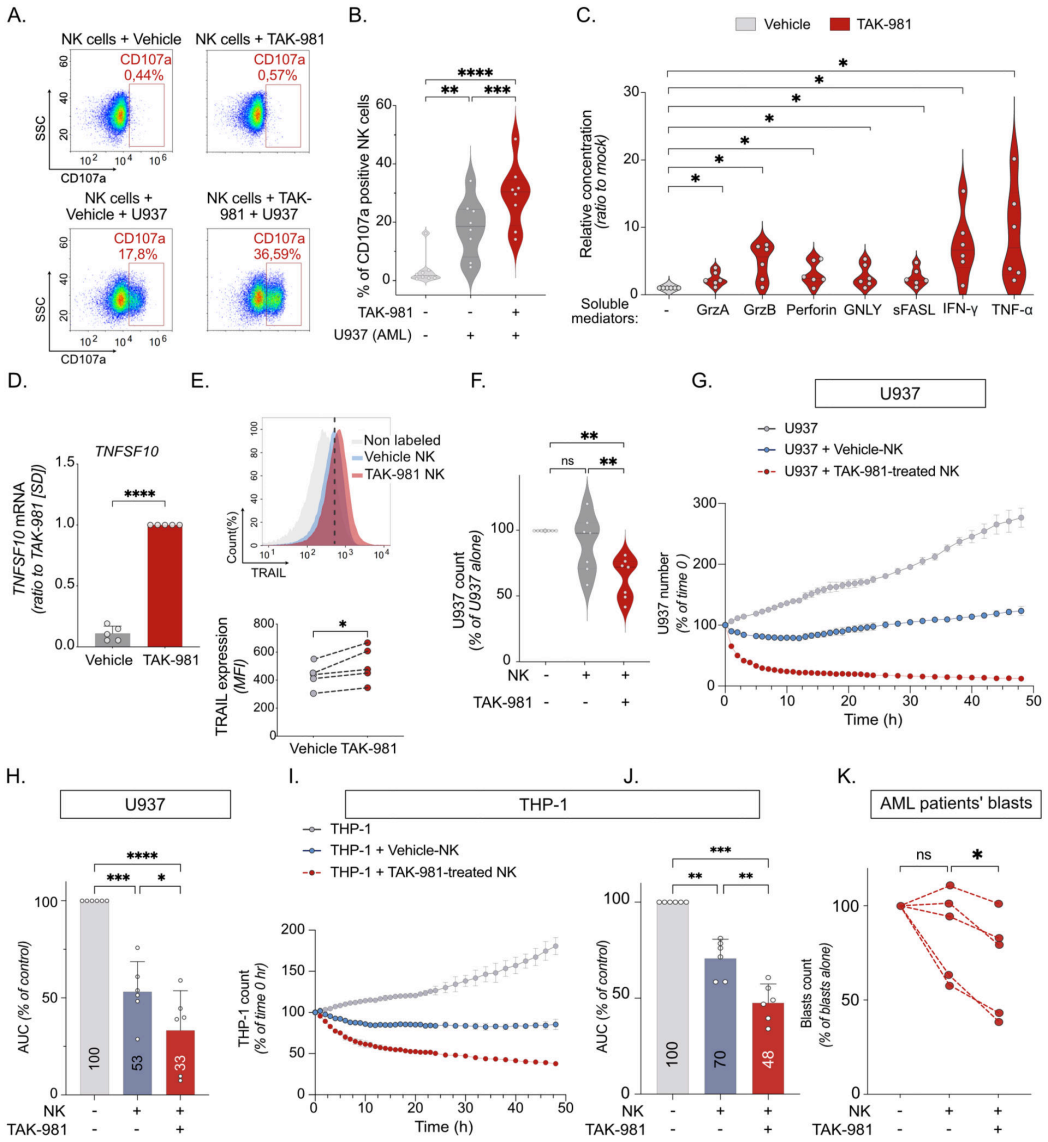
Inhibition of SUMOylation enhances NK cells cytotoxicity against cultured AML cells. **A. B**. Vehicle- and 100 nM TAK-981-treated NK cells were co-cultured or not for 4 h with U937 cells. **A**. Flow cytometry profiles showing the percentage of CD107a positive NK cells. **B**. Quantification of the percentage of CD107a positive NK cells. (n=8 donors, RM one-way ANOVA test). **C**. LEGENDplex human NK panel was performed on supernatant of NK cells pre-treated or not with 100 nM TAK-981 for 24 h and co-cultured for 4 h with U937 cells (E:T ratio 1:1). Relative quantity of each cytokine was normalized to vehicle-treated NK cells condition (n=6 donors, paired t-test). **D**. mRNA expression of *TNFSF10* in NK cells treated or not with 100 nM TAK-981 for 24 h (n=4 donors, paired t-test). **E**. The expression of TRAIL on the surface of NK cells treated with TAK-981 for 24 h was measured by flow cytometry (upper panel) and presented as relative MFI (lower panel, n=5 donors, paired t-test). **F**. U937-LucZsGreen cells were co-cultured for 4 h with NK cells vehicle - or TAK-981-treated for 24 h. Relative number of U937 cells was measured by flow cytometry (LucZsGreen fluorescence) and normalized to U937 cells without NK cells co-culture (n= 7 donors, one-way ANOVA test). **G**. Real-time immune cell killing assay: purified NK cells were pre-treated or not with TAK-981 for 24 h, followed by co-culture with U937-LucZsGreen at E:T ratio of 4:1. Green fluorescence was followed for 48 h using Incucyte device. A representative experiment is shown (data presented as number of green fluorescent cells +/- SD of 2 technical replicates). **H**. U937 proliferation was calculated using the area under the curve for each co-culture condition (n= 6 donors, RM one-way ANOVA). **I**. Real-time immune cell killing assay: purified NK cells were pre-treated or not with TAK-981 for 24 h, followed by co-culture with THP-1-LucZsGreen at E:T ratio of 4:1. Green fluorescence was followed for 48 h using Incucyte device. A representative experiment is shown (data presented as number of green fluorescent cells +/- SD of 2 technical replicates). **J**. THP-1 proliferation was calculated using the area under the curve for each co-culture condition (n= 6 donors, RM one-way ANOVA). **K**. AML patients’ blasts (4 patients) purified from bone marrow were co-cultured for 4 h with NK cells (n=3 donors) vehicle- or TAK-981-treated for 24 h (E:T ratio 1:1). Relative number of blast cells was measured by flow cytometry and normalized to blast cells number without NK cells co-culture.

As CD69 is an Interferon-Stimulated-Gene (ISG)(31), its increase at the surface of NK cells could solely reflect TAK-981-induced IFN-I secretion and is not indicative of productive NK engagement with targeted cells. We thus assessed whether TAK-981 increased NK cells cytotoxicity. In the presence of U937 as target cells, the percentage of NK cells expressing on their surface CD107a, a degranulation marker, was higher when they were pre-treated with TAK-981 compared to vehicle (Figure 2A and 2B). Confirming enhanced degranulation, TAK-981 increased the release of granzyme, perforin and granulysin from NK cells cytotoxic granules (Figure 2C). It also increased NK cells’ ability to secrete cytotoxic cytokines, including TNF-α and IFN-γ (Figure 2C). Finally, TAK-981 induced the expression of *TNFSF10*, encoding for TRAIL, in NK cells (Figure 2D) and a subsequent increase of TRAIL at their surface (Figure 2E), which binds to its receptors (DR4 and DR5) on the surface of cancer cells to induce their apoptosis. Accordingly, TAK-981 significantly increased the ability of NK cells to kill both U937 (Figure 2F, 2G and 2H, Supplementary Table S5) and THP-1 cell lines (Figure 2I and 2J, Supplementary Table S5) as well as AML patients blasts *in vitro* (Figure 2K, Supplementary Table S6). Finally, we checked for the effect of TAK-981 on (i) TIGIT (inhibitory receptor) and DNAM1 (activating receptor), which compete for binding to PVR and Nectin-2 on cancer cells, and (i) LFA1, which binds to ICAM-1 and stabilizes NK interaction with their target cells (32). Although TAK-981 did not affect TIGIT and DNAM1 at the surface of NK cells, it induced a small, yet significant, increase in LFA-1 (Supplementary Figure S1). Altogether, this suggests that inhibition of SUMOylation activates NK cells and boosts their cytotoxicity towards AML cells.

### TAK-981 activates AML patients’ NK cells and *ex vivo* expanded NK, both *in vitro* and *in vivo*

**Figure 3:**
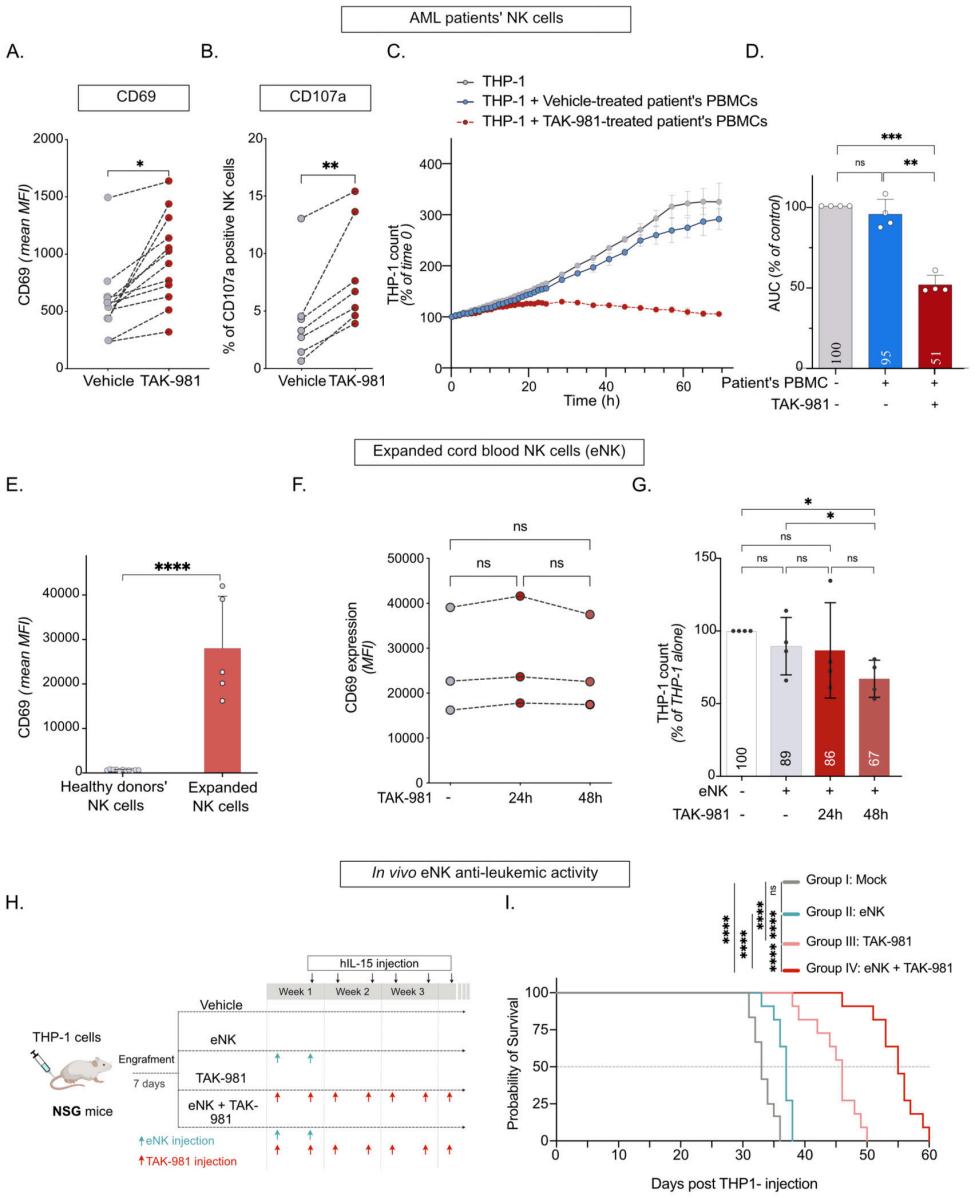
TAK-981 can both activate NK cells from AML patients and human eNK amplified from cord-blood. **A**. PMBC purified from AML patient’s blood or bone marrow were treated with 100 nM TAK-981 or vehicle for 24 h. Median fluorescent intensity of CD69 on NK cells (CD56^+^/CD3^-^ gating) treated with vehicle or TAK-981 is presented (n=12 patient samples, paired t test). **B**. Percentage of CD107a positive NK cells in AML patient’s PBMCs, treated or not with TAK-981 (n=7 patient samples, paired t-test). **C**. Real-time immune cell killing assay: AML patient’s PBMC (Patient #31 Supplementary Table S6) were pre-treated or not with TAK-981 for 24 h, followed by co-culture with THP-1-LucZsGreen at E:T ratio of 10:1. Green fluorescence was followed for 72 h using IncuCyte device. Number of THP-1-LucZsGreen cells is presented +/-SD of two technical replicates. **D**. THP-1 proliferation was calculated using the area under the curve for each co-culture condition (n= 4 patient’s PBMC, RM one-way ANOVA). **E**. Mean basal Median fluorescent intensity of CD69 in NK cells purified from healthy donors compared to eNK (mean of n=11 for healthy donors' NK, n=5 for eNK cells, unpaired t-test). **F**. eNK were treated with 100 nM TAK-981 for the indicated time. CD69 MFI of TAK-981-treated eNK cells are shown for 3 independent eNK preparations (RM one way ANOVA). **G**. THP-1 cells were co-cultured for 4 h with vehicle- or TAK-981-treated eNK cells for the indicated times. The number of THP-1 cells was normalized to THP-1 cells without eNK cells co-culture. Data are shown as mean +/- SD (n=4 donors, RM one-way ANOVA). **H**. Experimental design: NSG mice were injected intravenously with THP-1-LucZsGreen cells. After THP-1 cells engraftment, mice were randomized according to luminescence intensity and injected twice with eNK cells (day 7 and 10), followed by treatment with TAK-981 (15 mg/kg I.V.) and human recombinant IL-15 (hIL-15, 0.25 µg/mouse I.P.). For the rest of the experiment, mice were injected twice a week with TAK-981 and hIL-15. **I**. Overall survival was estimated in each group and compared with Kaplan-Meier method and log-rank test (n=11 mice/group).

TAK-981 potentiated the activation and degranulation capacity of NK cells present in PBMCs from AML patients’, as measured by the increased expression at their surface of CD69 (Figure 3A) and CD107a (Figure 3B), respectively. TAK-981 also enhanced the cytotoxicity of AML patient’s PBMC using THP1 as target cells (Figure 3C, D). We then turned to NK cells expanded from cord blood (eNK), which are used for adoptive transfer in many clinical trials, including in AML (3,5). Since eNK cells are amplified in the presence of IL-2 and IL-15, the levels of CD69 at their surface was higher than the basal level of expression on non-amplified NK cells from healthy blood donors (Figure 3E) and TAK-981 did not further increase its expression (Figure 3F). Nevertheless, TAK-981 induced an increase in their cytotoxicity after 48 h of treatment (Figure 3G). Immunodeficient NSG mice grafted with THP-1 cells were injected with eNK cells and mice were treated or not with TAK-981 (Figure 3H). The injection of eNK cells failed to significantly prolong mice survival. TAK-981, on its own, significantly prolonged mice survival as we previously described (15). The maximal effect on survival was observed when mice were both injected with eNK cells and treated with TAK-981 (Figure 3I). Thus, targeting SUMOylation could be of therapeutic interest in AML to both restore the anti-leukemic activity of patient’s own NK cells and increase the cytotoxicity of allografted eNK cells.

### Targeting SUMOylation specifically induces interferon pathway genes in NK cells

**Figure 4:**
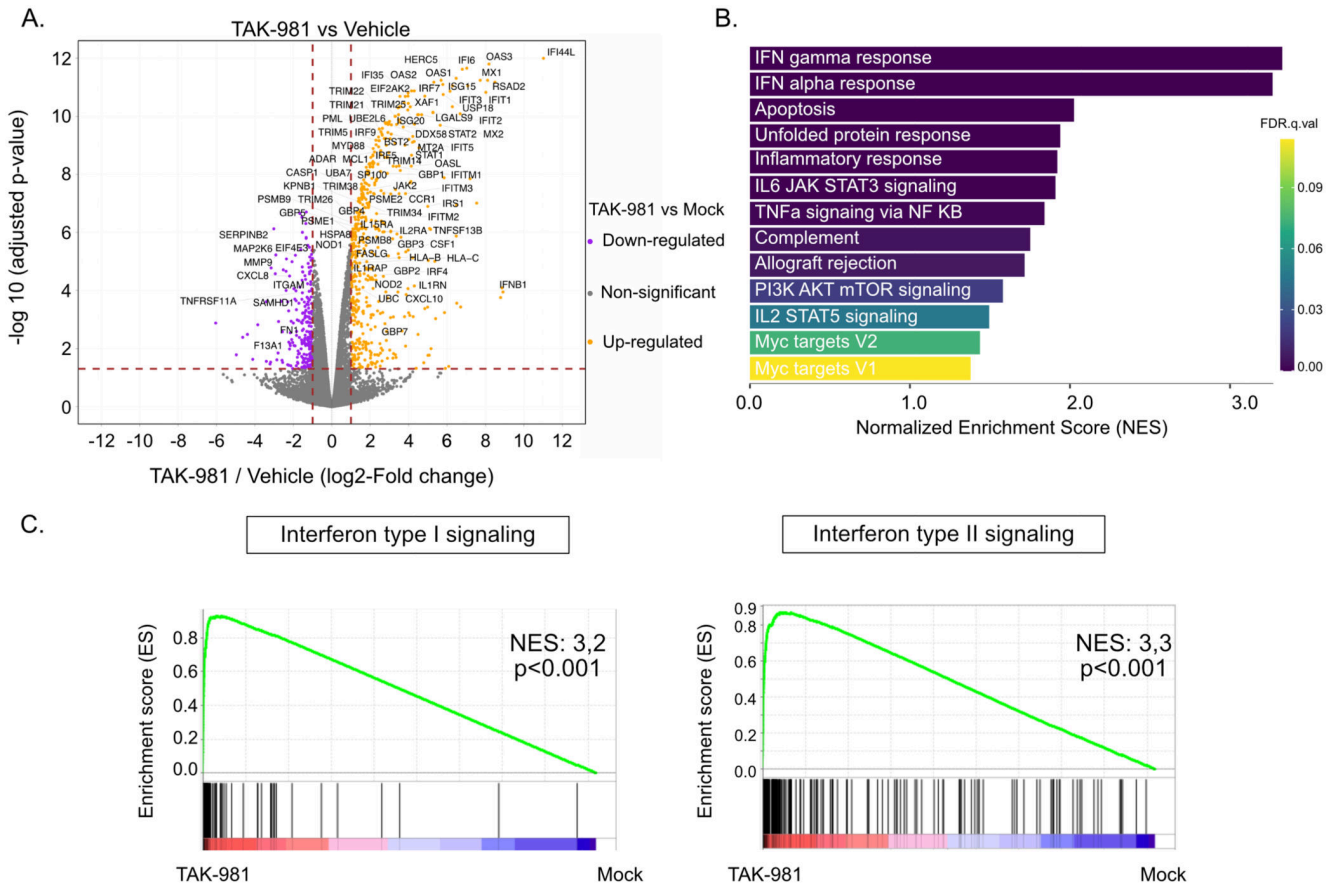
Targeting SUMOylation induces IFN-I pathway genes. **A**. Volcano plot of differentially expressed genes analyzed by RNA-Seq in NK cells purified from healthy donors’ blood (n=3) treated for 24 h with 100 nM TAK-981 compared to vehicle-treated controls. **B**. Gene Set Enrichment Analysis (GSEA) on Hallmark datasets on the RNA-Seq data presented in A. All pathways significantly enriched in TAK-981-compared to vehicle-treated cells are shown (abs(NES) > 1, p < 0.05 and FDR < 0.25). **C**. GSEA enrichment plot for the Interferon alpha and gamma signaling pathway in TAK-981-compared to vehicle-treated NK cells.

We then performed RNA-Seq experiments on FACS-sorted NK cells from 3 different healthy donors, treated *in vitro* with TAK-981 for 24 h. We identified 784 differentially expressed genes (log2 Fold change >1 or <-1, p<0.05), 536 being up-regulated and 248 down-regulated (Figure 4A, Supplementary Table S7). Gene set enrichment analysis revealed a strong enrichment of pathways linked to inflammation and immune response, in particular the interferon response (Figure 4B, 4C and Supplementary Table S7).

### SUMOylation represses interferon-pathway enhancers

**Figure 5:**
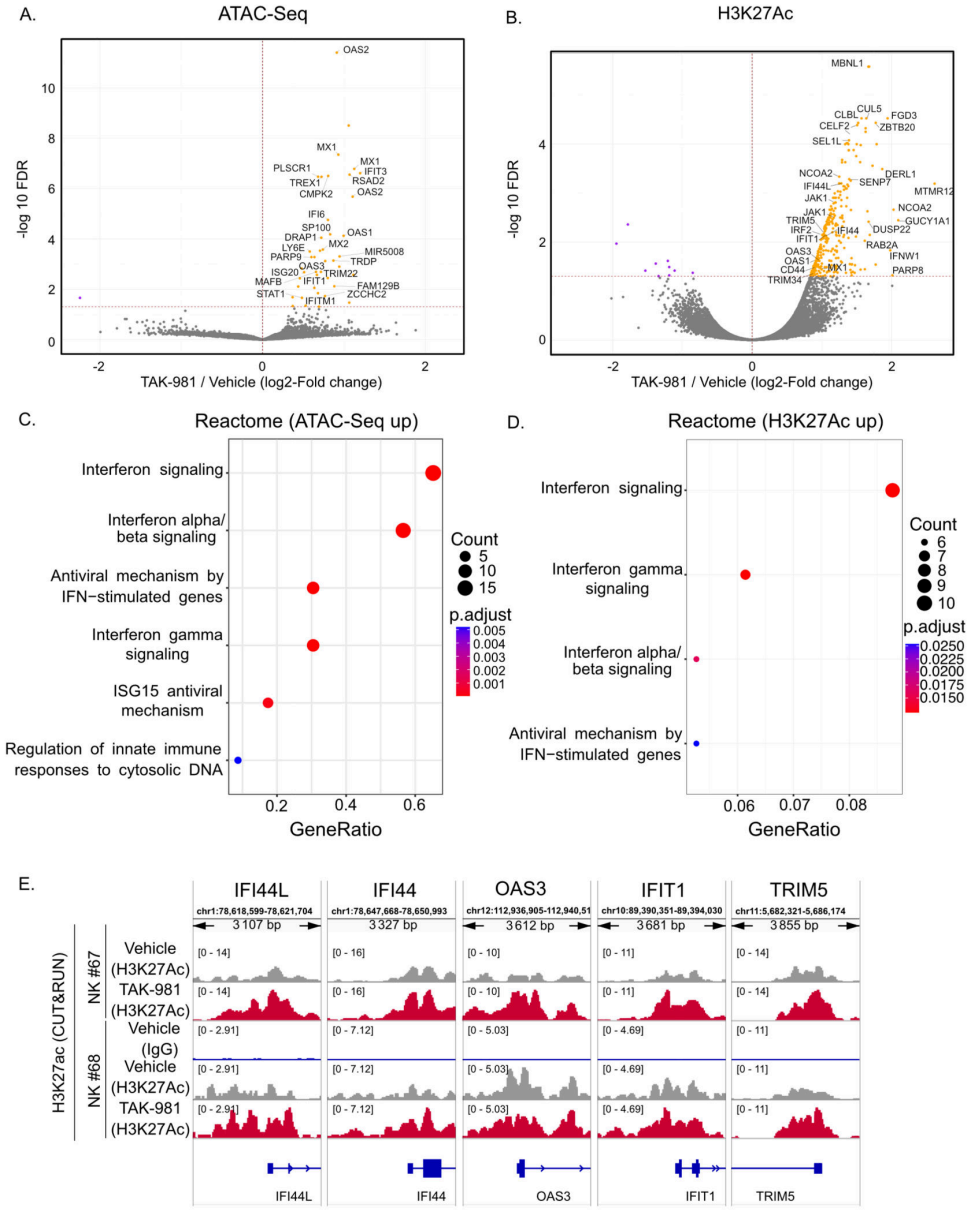
SUMOylation regulated IFN-I pathway genes enhancers activity and accessibility. **A**. Volcano plots of differentially accessible genomic regions analyzed by ATAC-Seq in NK cells purified from healthy donors’ blood (n=4 different donors) treated for 6 h with 100 nM TAK-981 compared to vehicle-treated controls. **B**. Volcano plots of genomic regions differentially enriched for H3K27ac mark analyzed by CUT&RUN in NK cells purified from healthy donors’ blood (n=2 different donors) treated for 6 h with 100 nM TAK-981 compared to vehicle-treated controls. **C**. Gene set enrichment analysis on the Reactome pathways of closest genes of genomic-regions showing increased accessibility upon TAK-981 treatment in A. **D**. Gene set enrichment analysis on the Reactome pathways of closest genes of genomic-regions showing increased H3K27ac upon TAK-981 treatment in A. **E**. H3K27Ac profiles at the *IFI44L, IFI44, IFIT1, TRIM5, OAS3* loci in NK cells purified from healthy donors and treated or not with TAK-981 for 6 h (n=2 donors). The data were scaled for each donor and represent average reads counts.

To decipher how SUMOylation controls IFN-I pathway activation, we analyzed the early effect of TAK-981 (6 h treatment) on (i) chromatin accessibility using ATAC-Seq and (ii) chromatin distribution of the histone mark H3K27ac, which marks active enhancers, with CUT&RUN (Figure 5). Inhibition of SUMOylation did not induce global changes in chromatin opening or H3K27ac deposition (Figure 5A, 5B). Despite the variability observed between the NK cells purified from 4 different donors, 42 genomic regions showed a statistically significant change in their accessibility upon TAK-981 treatment (Figure 5A). These peaks were largely located in the proximity of genes belonging to the IFN pathway (Figure 5C and Supplementary Figure S2). The effect of TAK-981 was more marked on H3K27ac with 320 genomic positions showing an increase in this mark of active enhancers (Figure 5C). These regions were also enriched in the vicinity of genes belonging to the interferon pathway (Figure 5D), such as *IFI44, IFI44L, IFIT1, TRIM5* and *OAS3* (Figure 5E and Supplementary Figure S2). Altogether, this suggests that TAK-981 rapidly and specifically activates the cis-regulatory regions of interferon pathway genes in NK cells, which likely explains their strong transcriptional up-regulation.

### TAK-981-induced NK cells activation requires IFN-I signaling

**Figure 6:**
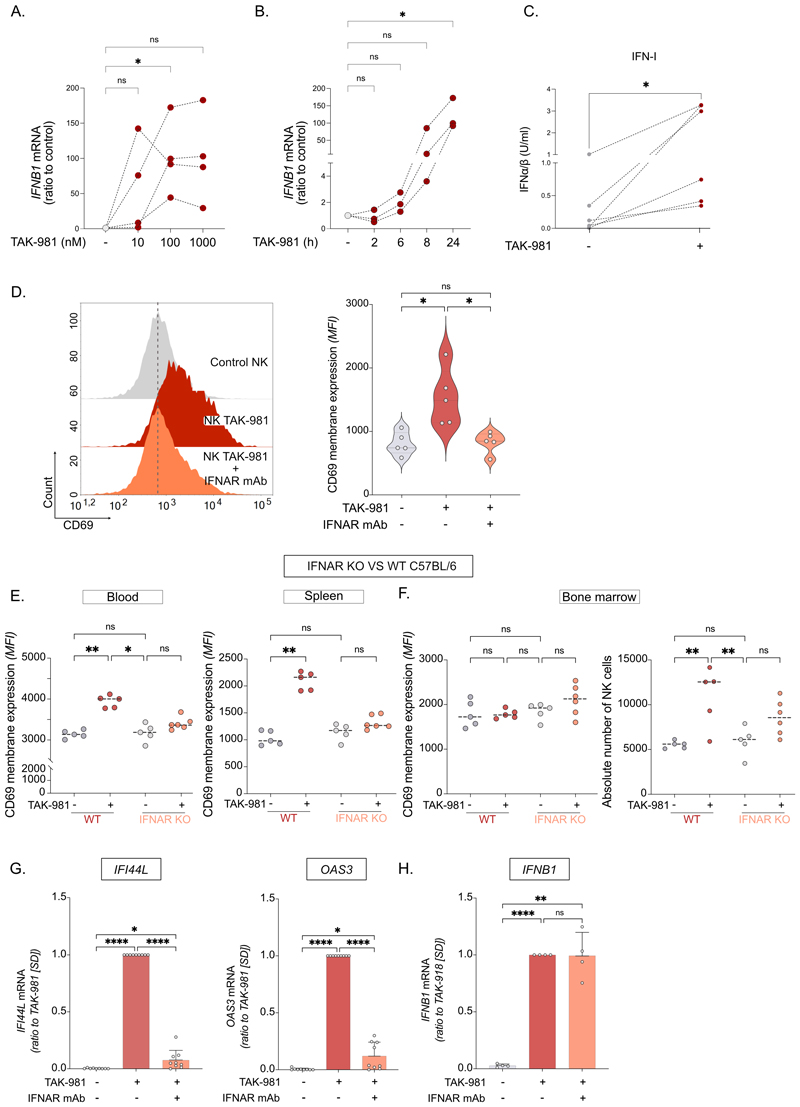
TAK-981-induced secretion of IFN-I is required for NK cells activation. **A**. Purified NK cells were treated for 24 h with TAK-981 at the indicated doses and analyzed by RT-qPCR for the expression of *IFNB1*. Data were normalized to non-treated cells (n=4 different healthy donors, RM one-way ANOVA). **B**. Purified NK cells were treated with TAK-981 (100 nM) for the indicated time and analyzed by RT-qPCR for the expression of *IFNB1*. Data were normalized to non-treated cells (n=3 different healthy donors, RM one-way ANOVA). **C**. Concentration of IFN-I in the supernatant of NK cells purified by FACS from healthy donors’ blood (n=7 donors) and treated with TAK-981 for 24 h. IFN-I was quantified with HEK blue IFN-α/β reporter cells (paired t-test). **D**. MFI of CD69 expression on the surface of NK cells treated or not with TAK-981 and IFNAR neutralizing monoclonal antibody for 24 h (left panel). Violin plot showing the quantification of CD69 expression (MFI) on the surface of NK cells treated or not with TAK-981 and IFNAR neutralizing monoclonal antibody for 24 h (right panel, n=5 donors, RM one-way ANOVA). **E. F**. WT and IFNAR KO C57BL/6 mice were injected intravenously with 15 mg/kg TAK-981 or vehicle. Mice were euthanized after 24 h and NK cells from blood, spleen and bone marrow were analyzed. **E**. CD69 expression levels on NK cells from blood and spleen of WT and IFNAR KO mice treated or not with TAK-981 (5 or 6 mice per group, RM one-way ANOVA). **F**. CD69 expression levels on NK cells and their absolute number per mice in bone marrow of WT and IFNAR KO mice treated or not with TAK-981 (5 or 6 mice per group, RM one-way ANOVA). **G. H**. mRNA expression levels of *OAS-3, IFI44L* (G) and *IFNB1* (H) in NK cells treated or not with TAK-981 and IFN-I receptor (IFNAR) neutralizing monoclonal antibody for 24 h (n=9 donors for *OAS-3* and *IFI44L*, n=5 donors for *IFNB1*. RM one-way ANOVA).

The *IFNB1* gene, encoding for Interferon-β, was found among the top-genes induced by TAK-981 in the RNA-Seq analysis (Figure 4A). To confirm the induction of *IFNB1*, we performed a TAK-981 dose-response on purified NK cells treated for 24 h. Although *IFNB1* was induced with 10 nM TAK-981 in NK cells from 2/4 donors, its induction was significant in all donors at 100 nM (Figure 6A). To determine the kinetics of *IFNB1* induction, we treated purified NK cells with 100 nM TAK-981 for different times. Although the induction of *IFNB1* was statistically significant only after 24 h, it started as early as 6 h after the beginning of the treatment (Figure 6B). Then, using a IFN-I-reporter cell line, we could show that NK cells treated with TAK-981 for 24 h secrete IFN-I in the medium (Figure 6C). To confirm the role of IFN-β secretion on TAK-981-induced autocrine activation of NK cells, NK cells were pre-incubated with an IFNAR-blocking antibody together with TAK-981 treatment. This largely decreased TAK-981-induced expression of CD69 on purified NK cells *in vitro* (Figure 6D). *In vivo*, TAK-981 induced the activation of NK cells in the blood and spleen of immunocompetent C57BL/6 mice after 24 h, as measured by the expression of CD69 at their surface (Figure 6E). This was not the case for NK cells from spleen and blood of mice knock-out for IFNAR (Figure 6F). Although TAK-981 did not activate NK cells in the bone marrow of wild-type mice, it significantly increased their numbers in this compartment in wild-type but not in IFNAR knock-out mice (Figure 6F). Secretion of IFN-I was necessary for the induction of Interferon Stimulated Genes (ISG) such as *OAS3* and *IFI44L*, as it was prevented by the addition, together with TAK-981, of the IFNAR-blocking antibody (Figure 6G), suggesting an autocrine activation mechanism. *IFNB1* induction was however not affected by IFNAR blocking (Figure 6H), suggesting that *IFNB1* is the primary gene induced by SUMOylation inhibition.

### NK cells are also activated *in trans* by IFN-β secreted mostly by myeloid cells upon inhibition of SUMOylation

**Figure 7:**
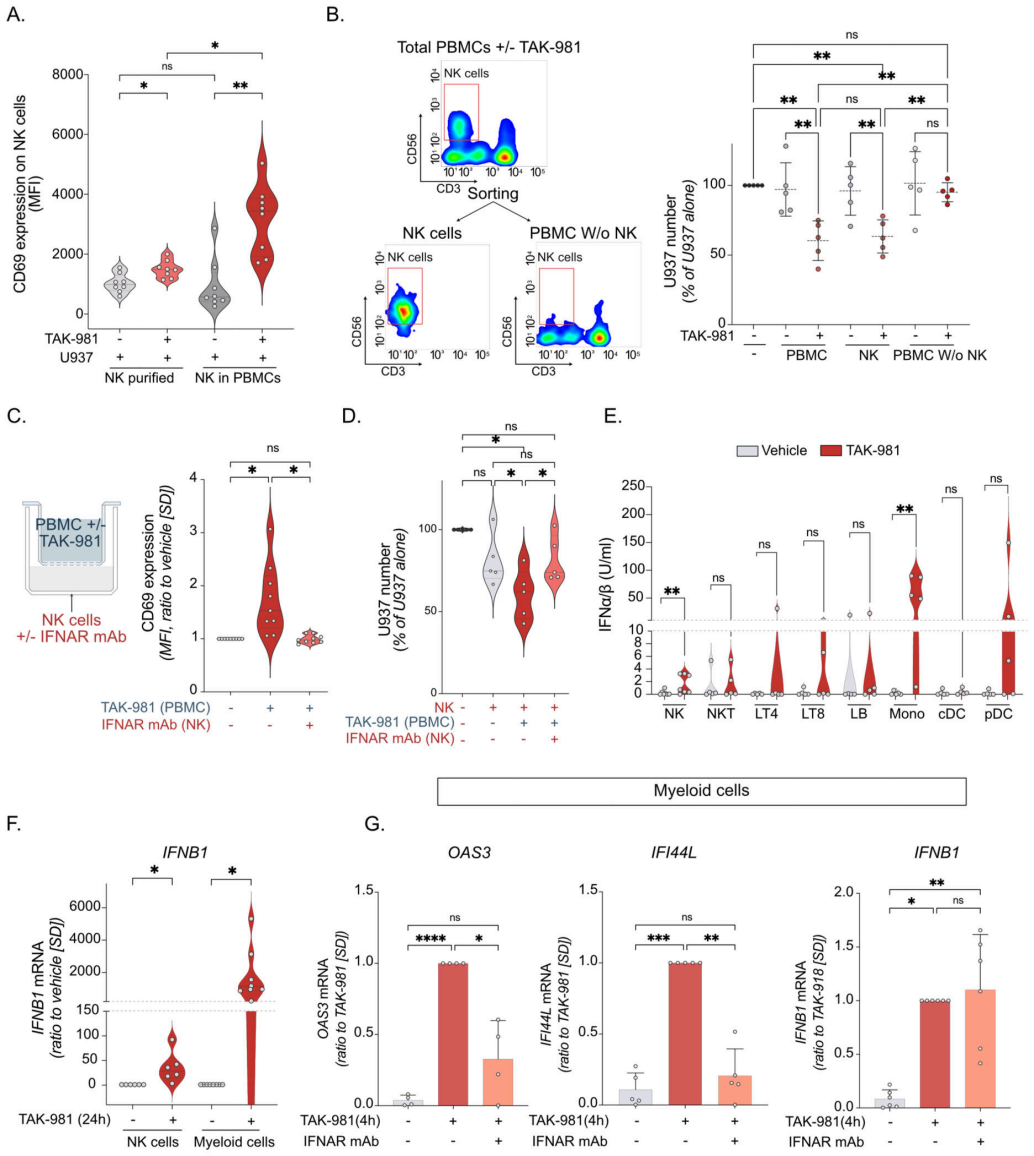
NK cells are activated *in trans* by IFN-I secreted by myeloid cells upon inhibition of SUMOylation. **A**. Purified NK cells or PBMC were treated with 100 nM TAK-981 for 24 h, followed by 4 h of co-culture with U937 (E:T ratio 1:1 for NK cells and 10:1 for PBMC). CD69 expression (MFI) on NK cells is presented (n=9 donors, RM one-way ANOVA). **B**. Total PBMC purified from healthy donors’ blood were treated with TAK-981 for 24 h followed by FACS to separate NK cells (CD56+ CD3) and NK-depleted PBMCs. Sorted populations or total PBMC where co-cultured for 4 h with U937-LucZsGreen cells. Relative number of U937 cells was measured by flow cytometry (LucZsGreen expression) and represented as the percentage of U937 cells without NK cells co-culture (n=5 donors, RM one-way ANOVA). **C**. PBMC were treated with TAK-981 for 24 h, washed and transferred in the upper chamber of a transwell. Purified NK cells +/-IFNAR blocking monoclonal antibody were placed in the lower chamber. After 24 h, NK cells were co-cultured with U937-LucZsGreen cells for 4 h. CD69 expression was measured on the surface of NK cells (right panel, n=9 donors, RM one-way ANOVA test). **D**. Relative number of U937 cells after 4 h of co-culture with NK cells treated as in C (n=5 donors, RM one-way ANOVA). **E**. NK cells, NKT cells, CD4+T cells (LT4), CD8+T cells (LT8), B-lymphocytes (LB), monocytes (Mono), classical dendritic cells (cDC) and plasmacytoid dendritic cells (pDC) were FACS-sorted and treated with TAK-981 for 24 h. IFN-I concentration was quantified in the supernatant with HEK Blue IFN-α/β reporter cells (n=7 donors for NK cells, n=5 donors for the rest, paired t-test). **F**. Relative expression of *IFNB1* mRNA in NK cells vs CD33+ myeloid cells purified from healthy donors’ blood and treated for 24 h with TAK-981. (n=6 donors for NK cells, and n=8 donors for CD33+ myeloid cells, paired t-test). **G**. Relative expression of *OAS-3, IFI44L* and *IFNB1* mRNA in purified CD33+ myeloid cells after 4 h treatment with TAK-981 +/- IFNAR blocking monoclonal antibody (n=4 donors for *OAS-3* gene, n=5 donors for *IFI44L* gene, n=5 donors for *IFNB1*, one-way ANOVA).

To assess whether NK cells could also be activated *in trans* by TAK-981, we compared its effect on purified NK cells and NK cells present within human PBMC. TAK-981 induced a stronger increase in CD69 at the surface of NK present within PBMCs than of purified NK cells (Figure 7A). To confirm that NK cells are indeed the main cells responsible for TAK-981-induced cytotoxicity towards AML cells, we performed cytotoxicity experiments with NK sorted after PBMCs treatment with TAK-981. NK cells purified from TAK-981-treated PBMC had equivalent cytotoxicity as total PBMCs and their depletion from PBMCs prevented U937 lysis (Figure 7B). We then performed transwell experiments using PBMCs +/-TAK-981 in the upper chamber and NK cells in the lower. This showed that soluble factors secreted by TAK-981-treated PBMC can activate NK cells (Figure 7C) and increase their cytotoxicity (Figure 7D). Moreover, the blocking IFNAR antibody prevented NK cells activation by these soluble factors, suggesting a main role for secreted IFN-I in NK cells activation *in trans* (Figure 7C, D). To identify, which immune cells, in addition to NK cells, are producing IFN-I upon inhibition of SUMOylation, T lymphocytes, B lymphocytes, NK cells, monocytes and dendritic cells were FACS-sorted from the PBMC of 5 different healthy blood donors. Purified cells were then treated *in vitro* with TAK-981 and IFN-I secretion was monitored using an IFNα/β reporter cell-line. Although most immune cell types were able to produce IFN-I upon TAK-981 treatment, at least for some of the donors, myeloid cells (monocytes and dendritic cells) were the strongest producers of IFN-I (Figure 7E). *IFNB1* was induced by TAK-981 to a mean of 1800-fold in purified myeloid cells (CD33+) compared to a mean of 35-fold in NK cells (Figure 7F). TAK-981 also strongly induced ISGs such as *OAS3* and *IFI44L* and *IFNB1* in myeloid cells after only 4 h. Similar to NK, blocking IFNAR strongly reduced *OAS3* and *IFI44L* induction but did not affect *IFNB1* (Figure 7G). Altogether, this suggests that inhibition of SUMOylation can also activate NK cells *in trans*, through the induction of IFN-I secretion, mostly by myeloid cells.

### SUMOylation represses *IFNB1* through a non-canonical pathway

**Figure 8:**
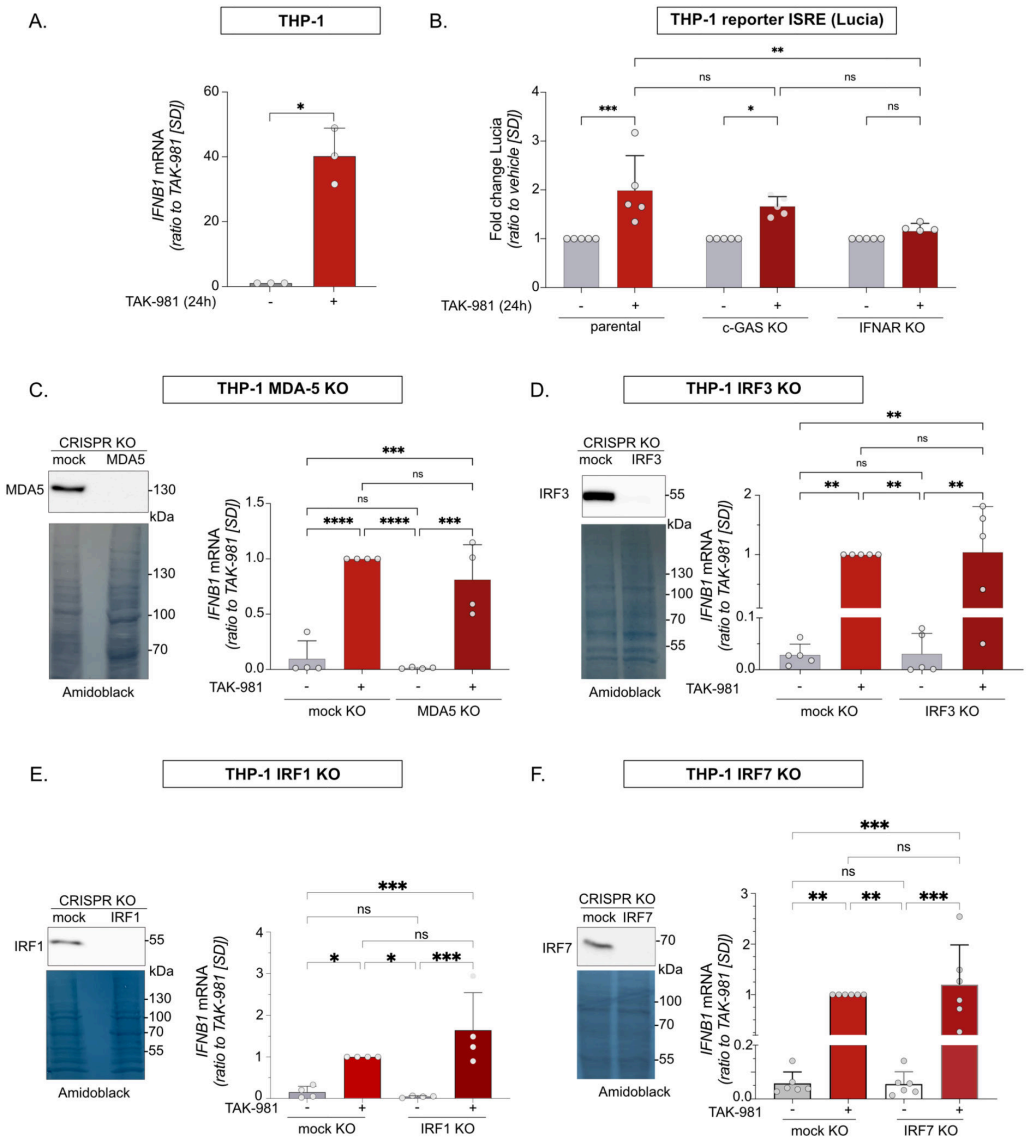
Inhibition of SUMOylation activates *IFNB1* through a non-canonical interferon pathway. **A**. Relative expression of *IFNB1* mRNA in THP-1 cells treated with 100 nM TAK-981 for 24 h (n=3, paired t-test). **B**. ISRE activation in THP1-ISRE-lucia parental, cGAS KO, and IFNAR KO treated with 100 nM TAK-981 or vehicle for 24 h. Luminescence data are presented as a ratio to vehicle-treated cells (n=5, one-way ANOVA). **C. D. E. F. Left panels:** CRISPR-Cas9-mediated knockouts of MDA5 (C), IRF3 (D), IRF1 (E), IRF7 (F) or control gRNA in THP-1 cells were analyzed for protein expression using anti-MDA5, IRF1, IRF3 and IRF7 antibodies respectively. Amido-black staining was used as loading control. **Right panels:** *IFNB1* mRNA expression levels in THP-1-MDA5-KO (C), THP-1-IRF3-KO (D), THP-1-IRF1-KO (E) and THP-1-IRF7-KO cells (F) treated with 100 nM TAK-981 or vehicle for 24 h (n=4 for MDA5, n=5 for IRF3, n=5 for IRF1, n=6 for IRF7, one-way ANOVA).

As TAK-981 strongly induced *IFNB1* in monocytes, we used the THP-1 monocytic cell line to address the underlying molecular mechanisms. TAK-981 indeed induced *IFNB1* expression after 24 h in this cell line (Figure 8A). TAK-981 also induced a reporter gene under the control of an ISRE (Interferon Stimulated Response Element), which is present in numerous ISGs (Figure 8B). Knock out of cGAS, a sensor of cytosolic DNA, which activates STING and, in turn, *IFNB1* (33), did not significantly affect the induction of the ISRE-reporter by TAK-981. On the contrary, deleting IFNAR prevented TAK-981-induced activation of the reporter (Figure 8B). This suggested that the activation of the ISRE-reporter observed in wild-type cells was indeed the consequence of IFN-β secretion and not a direct activation of ISRE-binding transcription activators by TAK-981. To understand how TAK-981 activates *IFNB1*, we knocked out its best-characterized upstream activators. Knock-out of MDA5 did not affect induction of *IFNB1* by TAK-981, excluding a role for the production of double-stranded RNA (dsRNA) in this process (Figure 8C). Deletion of IRF1 (Figure 8D), IRF3 (Figure 8E) and IRF7 (Figure 8F) also did not prevent *IFNB1* induction by TAK-981. Altogether, this suggests that the canonical interferon pathway proteins are not essential for TAK-981-induced *IFNB1* expression.

## Discussion

NK cells are increasingly considered as attractive therapeutic tools for cancer therapies either through the activation of patient’s own NK cells or by adoptive transfer of *in vitro* expanded NK cells (34). Here, we show that NK cells activation is under the control of the SUMO pathway and targeting SUMOylation with TAK-981 increases their anti-leukemic activity. These data are in line with previous work showing that TAK-981 increases human NK cells cytotoxicity against other tumor cell types (18). Inhibition of SUMOylation up-regulates more than 500 genes after 24 h, most of them belonging to the interferon pathway. Their activation is preceded by an increased accessibility and/or activation of their cis-regulatory regions. Secretion of IFN-I is required for the activation of NK cells and for the up-regulation of ISGs. In addition to this autocrine activation, NK cells are also activated by TAK-981-induced secretion of IFN-β by other immune cells, in particular myeloid cells. This paracrine activation likely plays a major role in NK cells activation *in vivo*.

In both NK and myeloid cells, the induction of the *IFNB1* gene by TAK-981 was not dependent on prior IFN-I secretion, suggesting it constitutes the primary target of TAK-981. The induction of IFN-I secretion was already described in various cell types, upon inhibition of SUMOylation, either by knocking-out the SUMO E1 or E2 enzymes (35,36) or by using SUMOylation inhibitors (17,18,37). This IFN-I secretion was shown to enhance the activation of various immune cells, including NK, contributing to the induction of an anti-tumoral immune response (17–19). How SUMOylation controls IFN-I pathway genes is however still largely unknown. Various Interferon response factors (IRFs) are SUMOylated and their SUMOylation was shown to limit their activity as measured by luciferase reporter assays (38–42). Using both reporter assays and CRISPR/Cas9 knock-out, we showed that IRF1, IRF3 and IRF7 are not essential for the activation of *IFNB1* by TAK-981. Other upstream regulators of *IFNB1* are SUMOylated. This is the case of MDA5, a sensor of double-stranded RNA, whose SUMOylation was shown to rather increase *IFNB1* expression (43,44). Here again, we show that knock-out of MDA5 has no effect on TAK-981 induced *IFNB1* expression. Similar results were found for c-GAS, a critical protein in the cytosolic DNA sensing pathway. Together with previous work showing that deletion of SAE2 induces an IFN-I response independently of IRF3, IRF7, STING and MAVS (35), this suggests that TAK-981-induced expression of *IFNB1* does not rely on the deSUMOylation of proteins of the canonical IFN-I activation pathways.

AML patients are generally treated with an immunosuppressive chemotherapy, which leads to bone marrow aplasia characterized by a loss of immune cells, including NK cells. At complete remission (CR), NK cell number increases but not to levels present in healthy donors. Moreover, although their degranulation capacities are restored at CR, their ability to produce cytokines remains low (45). As we demonstrate that TAK-981 can activate AML patient’s NK cells, it could be used after CR to increase NK cells activity towards residual AML cells. TAK-981 would thus have a dual anti-leukemic effect through a direct cytotoxic effect on AML cells as we and others have recently shown (15,16) and indirectly *via* the activation of patient’s own NK cells. The anti-leukemic action of NK cells would also be strengthened by the ability of SUMOylation inhibition to induce the expression of NK-activating ligands at the surface of cancer cells, as we demonstrated for ICAM-1 on AML cells (15) and as it was shown for PVR on Multiple Myeloma cells (46). Altogether, this would maximize the chances to control minimal residual disease (MRD), which is largely responsible for the high relapse rate in AML. Finally, we report that TAK-981 increases the cytotoxicity of *ex vivo* expanded NK cells from cord blood donors (eNK), both *in vitro* and *in vivo*. NK cells from cord blood can be efficiently expanded and matured *ex vivo*, making them ideal off-the-shelf cellular therapy (9,47,48). Although still in its early days, adoptive transfer of NK cells is increasingly considered for therapeutic use, including in AML, thanks to their anti-tumoral efficacy and their low toxicity (3,4,47–49). Intense research however aims at enhancing their antitumoral efficacy (9,10,50). Our work suggests that inhibiting SUMOylation with TAK-981 represents a promising new strategy to increase the anti-tumoral efficacy of NK cell-based therapies.

## Supplementary Material

1

2

3

4

5

6

7

8

9

## Data Availability

The RNA-Seq sequencing data are available on Gene Expression Omnibus with accession number GSE255279. The ATAC-Seq and CUT&RUN sequencing data are available on Gene Expression Omnibus with accession number GSE263923. Other data generated in this study are available from the Corresponding Author upon reasonable request.
