# Supplementary material for: Targeting SUMOylation triggers interferon-β-dependent activation of patient and allogenic Natural Killer cells in preclinical models of Acute Myeloid Leukemia
Source: Mol Cancer Ther. Author manuscript; Available in PMC 2025 Aug 15. (PMC7618005; doi:10.1158/1535-7163.MCT-25-0504)
Supplement: 1 [file EMS207354-supplement-1.pdf]

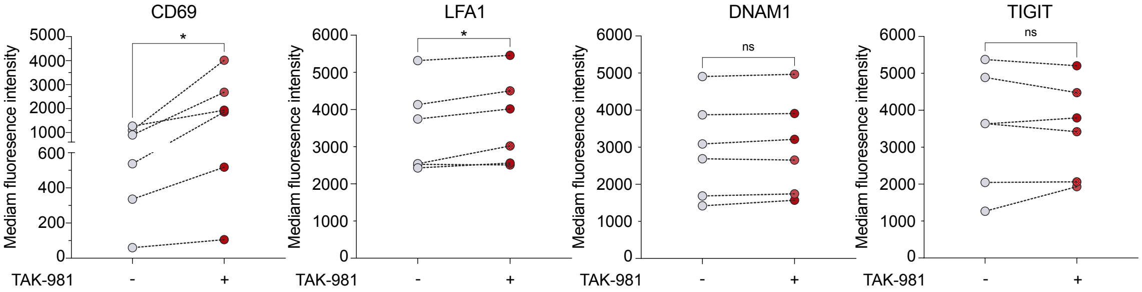

Supplementary Figure S1: TAK-981 induces CD69 and LFA-1 but not does not affect DNAM1 and TIGIT expression at the surface of NK cells. CD69, DNAM1, TIGIT and LFA-1 expression was measured by flow cytometry on NK cells present in PBMCs from healthy donors treated for 24 h with 100 nM TAK-981 (n=6, Paired student t-test).
