# Supplementary material for: Targeting SUMOylation triggers interferon-β-dependent activation of patient and allogenic Natural Killer cells in preclinical models of Acute Myeloid Leukemia
Source: Mol Cancer Ther. Author manuscript; Available in PMC 2025 Aug 15. (PMC7618005; doi:10.1158/1535-7163.MCT-25-0504)
Supplement: 2 [file EMS207354-supplement-2.pdf]

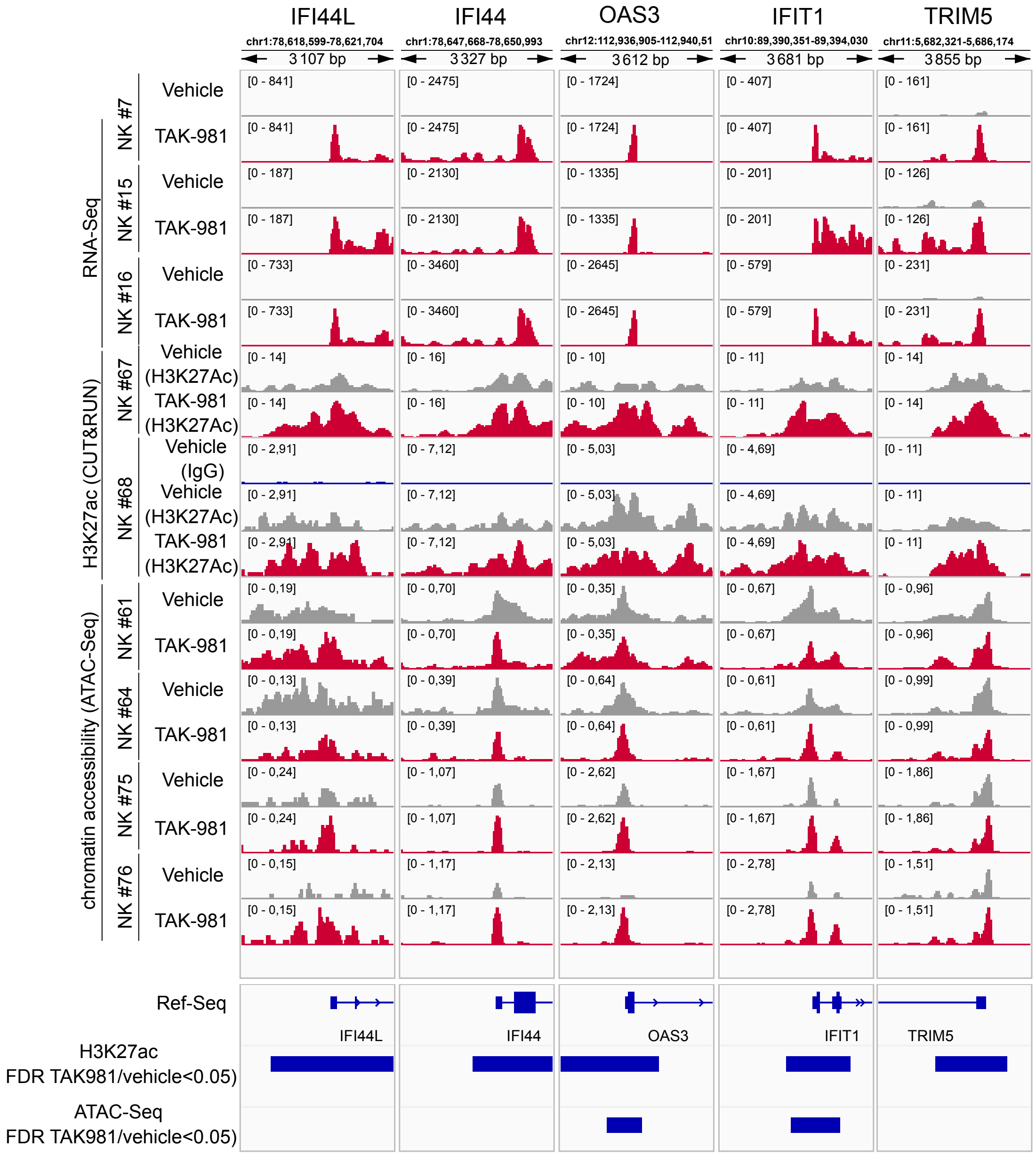

Supplementary Figure S2: SUMOylation regulated IFN-I pathway genes enhancers activity and accessibility. RNA-Seq, H3K27Ac CUT&RUN and ATAC-Seq profiles at the *IFI44L*, *IFI44*, OAS3, *IFIT1*, *TRIM5* in NK cells purified from healthy donors and treated or not with TAK-981 for 6 h (n=3 donors for RNA-Seq, n=2 donors for H3K27ac and n=4 donors for ATAC-Seq). The peaks identified as differentially enriched between vehicle- and TAK-981-treated NK cells in the H3K27ac CUT&RUN or in the ATAC-Seq are indicated.
