# Supplementary material for: Targeting SUMOylation triggers interferon-β-dependent activation of patient and allogenic Natural Killer cells in preclinical models of Acute Myeloid Leukemia
Source: Mol Cancer Ther. Author manuscript; Available in PMC 2025 Aug 15. (PMC7618005; doi:10.1158/1535-7163.MCT-25-0504)
Supplement: 3 [file EMS207354-supplement-3.pdf]

Supplementary Table S1: antibodies used for flow cytometry. The name of the targeted protein, the fluorochrome and the reference are indicated

	Target	Fluorochrome	Reference
Human	7-AAD	_	Miltenyi 130-111-568
	CD107a	FITC	Miltenyi 130-111-620
	CD107a	APC	Miltenyi 130-111-847
	CD11b	APC	Miltenyi 130-110-554
	CD11c	PE-Vio 770	Miltenyi 130-113-588
	CD123	APC-Vio 770	Miltenyi 130-115-267
	CD14	PE	Miltenyi 130-110-519
	CD16	PE	Miltenyi 130-113-393
	CD16	VioBlue	Miltenyi 130-113-396
	CD19	APC	Miltenyi 130-113-642
	CD19	APC-Vio 770	Miltenyi 130-113-643
	CD253 (TRAIL)	PE-Vio 615	Miltenyi 130-119-286
	CD3	FITC	Miltenyi 130-113-138
	CD3	PE-Vio 615	Miltenyi 130-114-520
	CD3	APC	Miltenyi 130-113-135
	CD33	PerCP-Vio 700	Miltenyi 130-111-023
	CD4	APC-Vio 770	Miltenyi 130-113-223
	CD45	APC	Miltenyi 130-110-633
	CD45	FITC	Miltenyi 130-110-631
	CD45	VioGreen	Miltenyi 130-110-638

	CD56	APC-Vio 770	Miltenyi 130-114-548
	CD56	FITC	Miltenyi 130-114-549
	CD56	PE Vio 770	Miltenyi 130-113-313
	CD69	PE	Miltenyi 130-112-613
	CD8	PE	Miltenyi 130-110-816
	HLA-DR	FITC	Miltenyi 130-111-941
Murine	CD11b	APC	Miltenyi 130-109-286
	CD122 (IL-2Rb)	FITC	Miltenyi 130-102-481
	CD19	APC-Vio 770	Miltenyi 130-111-886
	CD3	PerCP-Vio 700	Miltenyi 130-120-826
	CD45	PE-Vio 770	Miltenyi 130-110-799
	CD69	PE	Miltenyi 130-115-575
	NKp46	APC-Vio 770	Miltenyi 130-112-361
