# Supplementary material for: Targeting SUMOylation triggers interferon-β-dependent activation of patient and allogenic Natural Killer cells in preclinical models of Acute Myeloid Leukemia
Source: Mol Cancer Ther. Author manuscript; Available in PMC 2025 Aug 15. (PMC7618005; doi:10.1158/1535-7163.MCT-25-0504)
Supplement: 4 [file EMS207354-supplement-4.pdf]

Supplementary Table S2: Primers sequences for ATAC-seq Tagmented DNA amplification

Name	Primer	Sequence
Ad1	Ad1_noMX	AATGATACGGCGACCACCGAGATCTACACTCGTCGGCAGCGTCAGATGTG
Ad2	N707	CAAGCAGAAGACGGCATACGAGATGTAGAGAGGTCTCGTGGGCTCGGAGATGT
	N708	CAAGCAGAAGACGGCATACGAGATCCTCTCTGGTCTCGTGGGCTCGGAGATGT
	N713	CAAGCAGAAGACGGCATACGAGATATCACGACGTCTCGTGGGCTCGGAGATGT
	N714	CAAGCAGAAGACGGCATACGAGATACAGTGGTGTCTCGTGGGCTCGGAGATGT
