# Supplementary material for: Targeting SUMOylation triggers interferon-β-dependent activation of patient and allogenic Natural Killer cells in preclinical models of Acute Myeloid Leukemia
Source: Mol Cancer Ther. Author manuscript; Available in PMC 2025 Aug 15. (PMC7618005; doi:10.1158/1535-7163.MCT-25-0504)
Supplement: 5 [file EMS207354-supplement-5.pdf]

Supplementary Table S3: Primers sequences used for RT-qPCR.

**Target gene**	**Forward primer (5’ -> 3’)**	**Reverse primer (5’ -> 3’)**
IFNB1	ATGGTCAATGCGGCGTCCTC	TGTGGCAATTGAATGGGAGGC
TNFSF10	GGCTGCCTGGCTGACTTACA	GATCACGATCAGCACGCAGG
OAS3	AGCCCAGGTTCAGGTCTACCC	AGTGTTTTCCCATCTCTGCTCTGC
IFI44L	GCAAAAATTGAGGGCACAGGGG	ACAAATGAGCTCAGTGGCTTGG
GAPDH	CTGCACCACCAACTGCTTAG	AGGTCCACCACTGACACGTT
