# Supplementary material for: Targeting SUMOylation triggers interferon-β-dependent activation of patient and allogenic Natural Killer cells in preclinical models of Acute Myeloid Leukemia
Source: Mol Cancer Ther. Author manuscript; Available in PMC 2025 Aug 15. (PMC7618005; doi:10.1158/1535-7163.MCT-25-0504)
Supplement: 6 [file EMS207354-supplement-6.pdf]

Supplementary Table S4: gRNA sequences for CRISPR/KO. Sequences of gRNA used to knock out MDA5, IRF1, IRF3 and IRF7 in THP1 cells.

Target	gRNA sequence (5’->3’)
MDA5	CACCGCGAATTCCCGAGTCCAACCA
IRF1	CTTGGCAGCATGCTTCCATGGG
IRF3	AAACCACATACTGGGCAGTGAGCGC
IRF7	AAACGGAAGCACTTCGCGCGCAAGC
